# CACHD1: A new activity-modifying protein for voltage-gated calcium channels

**DOI:** 10.1080/19336950.2019.1600968

**Published:** 2019-04-13

**Authors:** Gary J. Stephens, Graeme S. Cottrell

**Affiliations:** School of Pharmacy, University of Reading, Reading, UK

Understanding the mechanisms that regulate the trafficking and expression of voltage-gated calcium channels (VGCCs) at presynaptic and potentially postsynaptic plasma membrane microdomains is of paramount importance. VGCCs are subdivided into two main families: high voltage-activated calcium channels (HVACCs) and low voltage-activated calcium channels (LVACCs) (reviewed [1]). HVACCs comprise Ca_V_1.1–4 (L-type current), Ca_V_2.1 (P/Q-type current), Ca_V_2.2 (N-type current) and Ca_V_2.3 (R-type current) VGCCs, whereas LVACCs comprise the Ca_V_3 (T-type current) family. The expression, assembly, localization and biophysical properties of HVACCs can be modulated by α_2_δ and β auxiliary subunits. However, the modulation of Ca_V_3 channels by other proteins has remained less well defined. α_2_δ and β subunits are not considered to be auxilliary subunits for Ca_V_3 subunits and consensus opinion is that Ca_V_3 can form fully functional channels alone (reviewed [2]). We have recently identified CACHD1 (Ca^2+^ channel and chemotaxis receptor (cache) domain containing protein 1), a protein with structural similarities to the α_2_δ family, as a modulator of Ca_V_3 VGCC activity in expression systems and in native neurons [3]. Our study adds to other recently defined modulators of Ca_V_3 subunits which include endogenous molecules such as the actin binding protein kelch-like 1 (KLHL1), the Stac adaptor protein 1 (Stac1), the endoplasmic reticulum integral membrane protein, calnexin and the ubiquitin-specific protease, USP5 (reviewed [4]).

**Figure 1.**
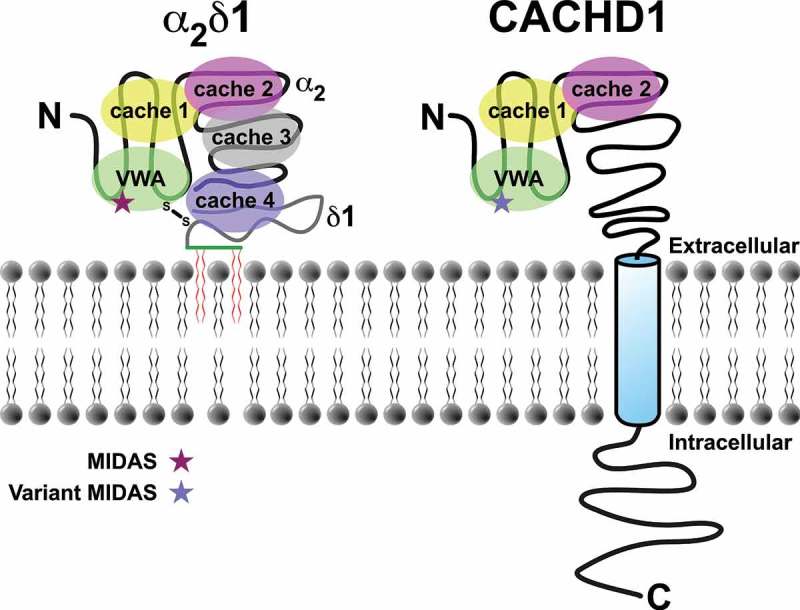
**Schematic comparison of the major domains and structural motifs of α_2_δ1 and CACHD1. (Left)** α_2_δ1 is translated as a single polypeptide chain that is proteolytically processed into two chains (α_2_ and δ), which are covalently connected by a disulphide bridge. The δ chain is further processed to tether the protein to the plasma membrane, reportedly via a glycophosphatidylinositol anchor (reviewed [2]). Crystallographic studies reveal that the large extracellular facing portion of α_2_δ1 contains a von Willebrand Factor A (VWA) domain and four cache domains [9]. Within the VWA domain, α_2_δ1 possesses a consensus metal ion-dependent adhesion site (MIDAS) motif that is important for its interaction with pore-forming α_1_ subunits. (**Right**) CACHD1 is a single polypeptide chain with a large extracellular N-terminal domain, a single transmembrane domain and an intracellular C-terminal tail. Similar to α_2_δ1, the extracellular region of CACHD1 contains a single VWA domain. Currently, CACHD1 has two predicted cache domains and contains a variant MIDAS motif within its VWA domain.

Primary sequence analysis reveals that although CACHD1 has limited sequence identity to α_2_δ subunits, it similarly contains a VWA (von Willebrand Factor A) domain and putative bacterial chemosensory-like cache domains (Figure 1). However, there are a number of important differences compared to α_2_δ subunits that will potentially affect CACHD1 function. For example, CACHD1 contains fewer predicted glycosylation sites, a variant metal ion-dependent adhesion site (MIDAS) motif (D^234^xGxS) within the VWA domain and a variant gabapentin binding site (R^213^-S-R). Of potential importance, CACHD1 is predicted to be a single transmembrane protein with a large intracellular C-terminal tail, unlike α_2_δ, which is reportedly a glycophosphatidylinositol (GPI)-anchored protein (reviewed [2]). In our study, we demonstrated that CACHD1 promoted the cell surface localization of Ca_V_3.1 in a heterologous expression system [3]. Furthermore, using proximity ligation assays, we showed that CACHD1 and Ca_V_3.1 are <40 nm apart at the cell surface, supporting the formation of CACHD1∙Ca_V_3.1 complexes at the plasma membrane. Our electrophoretic analyses indicated that CACHD1 migrated slower than its predicted molecular mass of 142 kDa, migrating at approximately ~170 kDa, possibly indicating post-translational glycosylation. Human CACHD1 was shown to cause clear effects on human Ca_V_3.1, Ca_V_3.2 and Ca_V_3.3 subunits, increasing peak current density with a corresponding increase in maximal conductance. In contrast, expression of α_2_δ1 had no effect on T-type current under the same conditions. CACHD1 increased the open probability of the Ca_V_3.1 channel, pointing to mechanistic effects on the α_1_ subunit. We further demonstrated functional effects of CACHD1 on native T-type current in rat hippocampal neurons. CACHD1 transfected neurons fired at a higher frequency than control neurons, an effect that was prevented by the selective T-type current blocker TTA-P2. Furthermore, CACHD1 caused a significant increase in rebound firing frequency, a property mediated predominantly by T-type current; TTA-P2 similarly reversed this effect. Together, these data support a role for CACHD1 in increasing neuronal T-type current, which in turn promotes an increase in action potential firing frequency and neuronal excitability. The future study of neurons from CACHD1 knockout mice will further advance knowledge of the physiological role of CACHD1.

A subsequent report has also identified a role for CACHD1 as a modulator of other VGCC subtypes, reporting effects on Ca_V_2.2 [5]. Together, these studies suggest that CACHD1 may have broader therapeutic potential. However, although CACHD1 caused a significant increase in Ca_V_2.2 current, this modulation was not as profound as seen for α_2_δ1; indeed, co-expression of CACHD1 caused a reduction in α_2_δ1-mediated actions on Ca_V_2.2. These data indicate that CACHD1 may have a dominant negative-like effect on N-type calcium current and led to the suggestion that CACHD1 and α_2_δ1 may compete for the same structural motif on Ca_V_2.2 [5], unlike the situation for Ca_V_3 subunits. In our original studies, we did not detect any significant increase in Ca^2+^ current for human Ca_V_2.2 co-expressed with human CACHD1 [6], in contrast to effects reported for rat Ca_V_2.2 with zebrafish or rat CACHD1 [5]. Of further interest was that these CACHD1 orthologues had no effect on rat Ca_V_2.1 [5]. Therefore, it may be that CACHD1 effects are dependent on the species of both CACHD1 and the α_1_ subunit.

The α_2_δ MIDAS motif (DxSxS) within its VWA domain plays a major role in their modulation of VGCC function (reviewed [2]). Approximately 46% of VWA domain-containing proteins contain a conserved MIDAS motif [7]. CACHD1 has a variant MIDAS motif (D^234^xGxS). Of functional importance, structural studies of VWA domains and biochemical analysis of a family of proteins called copines revealed that a perfectly conserved MIDAS motif is not required for metal ion binding [7]. Due to the difficulties of predicting non-contiguous structural motifs, such as cache domains, from primary sequences, it remains undetermined if the downstream Thr and Asp residues in human CACHD1 contribute to a conserved MIDAS motif. However, one could speculate, based on our preliminary sequence alignments, that these residues are Thr^311^ and Asp^338^. Mutation of the Asp and Ser residues to Ala (AxAxA) in the α_2_δ1 MIDAS motif caused disruption of the interaction between α_2_δ1 and Ca_V_2.2 and this interaction was also prevented by mutation of Asp^122^ to Ala on Ca_V_2.2 [5]; surprisingly, the D122A mutation did not prevent CACHD1-mediated enhancement of Ca_V_2.2 currents. This data may indicate that CACHD1 does not interact with Ca_V_2.2 in exactly the same way as α_2_δ1. Future biochemical and electrophysiological studies involving mutated forms of the CACHD1 variant MIDAS motif will shed light on its role in the regulation of the function of both CACHD1 and VGCCs. The electrophoretic mobility of rat CACHD1 was increased to its predicted level by treatment of whole cell lysates with the N-linked deglycosylating enzyme, PNGase F, indicating that rat CACHD1 is modified by N-linked glycosylation [5]. This study highlighted seven high potential N-linked glycosylation sites on rat CACHD1. Similar sequence analysis of human CACHD1 reveals one very high probability site (Asn^145^), five other high probability Asn residues, with an additional three lower potential sites. Future experimentation will reveal precisely which residues are glycosylated and the role of glycosylation in the function of human CACHD1.

To date, CACHD1 and the α_2_δ isoforms are the only mammalian proteins known to contain bacterial chemosensory-like cache domains. Cache domains are the most important extracellular sensors in prokaryotes (reviewed [8]) and may represent potential drug targets. The current predicted CACHD1 structure, including two predicted cache domains, is based on extant Uniprot data (Q5VU97). For α_2_δ1, the initial description of a single cache domain (Uniprot data, P54289) has been revised to two and most recently to four domains following the latest crystal structure at 3.6 Å [9]. A similar crystal structure will be required to determine the number of cache domains in CACHD1, but the presence of multiple cache domains adds another dimension to the possible functionality of both α_2_δ and CACHD1.

With regard to our recent work, CACHD1 is predicted to act mechanistically to increase Ca_V_3 channel open probability and promote T-type currents. Such physiological actions may have implications in targeting diseases involving aberrant neuronal firing. In particular, Ca_V_3 channels have been proposed as therapeutic targets to combat pain and epilepsy (reviewed [4]). It is of particular interest that CACHD1 transcripts are highly expressed in mouse dorsal root ganglia [10], key transducers of nociceptive information. However, the therapeutic potential of Ca_V_3-targeting drugs remains largely unrealized, as clinical drugs such as ethosuximide lack Ca_V_3 selectivity and an earlier agent, mibefradil, was withdrawn due to serious drug interaction issues. It will be of interest to identify the functions of the domains and sequence motifs present in CACHD1 responsible for its effects on T-type currents and the sequences within Ca_V_3 channels that may participate in the interaction with CACHD1. Targeting proteins such as CACHD1 that modulate Ca_V_3 function may provide an attractive alternative to the direct blocking of the α_1_ subunit pore and we propose that CACHD1 represents an important new protein with excellent potential for future therapeutic targeting.
